# Monocyte infiltration is an independent positive prognostic biomarker in vulvar squamous cell carcinoma

**DOI:** 10.1007/s00262-024-03755-w

**Published:** 2024-07-02

**Authors:** Ziena Abdulrahman, Kim E. Kortekaas, Marij J. P. Welters, Mariette I. E. van Poelgeest, Sjoerd H. van der Burg

**Affiliations:** 1 Department of Medical Oncology, Oncode Institute, Leiden University Medical Center, P.O. Box 9600, 2300 RC Leiden, The Netherlands; 2 Department of Gynecology, Leiden University Medical Center, Leiden, The Netherlands

**Keywords:** Tumor immune microenvironment, Immunotherapy, Vulvar squamous cell carcinoma, Prognostic biomarker

## Abstract

**Background:**

Vulvar squamous cell carcinoma (VSCC) arises after an HPV infection or the mutation of p53 or other driver genes and is treated by mutilating surgery and/or (chemo) radiation, with limited success and high morbidity. In-depth information on the immunological make up of VSCC is pivotal to assess whether immunotherapy may form an alternative treatment.

**Methods:**

A total of 104 patient samples, comprising healthy vulva (*n* = 27) and VSCC (*n* = 77), were analyzed. Multispectral immunofluorescence (15 markers) was used to study both the myeloid and lymphoid immune cell composition, and this was linked to differences in transcriptomics (NanoString nCounter, 1258 genes) and in survival (Kaplan–Meier analyses).

**Results:**

Healthy vulva and VSCC are both well infiltrated but with different subpopulations of lymphoid and myeloid cells. In contrast to the lymphoid cell infiltrate, the density and composition of the myeloid cell infiltrate strongly differed per VSCC molecular subtype. A relative strong infiltration with epithelial monocytes (HLADR^−^CD11c^−^CD14^+^CD68^−^CD163^−^CD33^−^) was prognostic for improved survival, independent of T cell infiltration, disease stage or molecular subtype. A strong infiltration with T cells and/or monocytes was associated with drastic superior survival: 5-year survival > 90% when either one is high, versus 40% when both are low (*p* < 0.001).

**Conclusion:**

A hot myeloid and/or lymphoid infiltrate predicts excellent survival in VSCC. Based on the response of similarly high-infiltrated other tumor types, we have started to explore the potential of neoadjuvant checkpoint blockade in VSCC.

**Supplementary Information:**

The online version contains supplementary material available at 10.1007/s00262-024-03755-w.

## Introduction

Vulvar squamous cell carcinoma (VSCC) can arise due to three distinct etiological pathways, each with differential clinical prognosis: driven by classical oncogenic mutations in TP53 (HPV^−^p53mut VSCC, poorest clinical outcome), driven by other somatic oncogenic mutations (HPV^−^p53wt, intermediate clinical outcome) or driven by a persistent infection with the oncogenic human papillomavirus (HPV^+^ VSCC, best clinical outcome) [1, 2]. The current standard of care for VSCC is surgery and/or (chemo) radiation, which is associated with high morbidity due to severe local or systemic side effects. Therefore, novel treatment strategies are urgently needed.

Immunotherapy is increasingly being explored as an alternative for current standard cancer treatments. Studies in other tumor types have shown the importance of a pre-existing well-coordinated immune response for the prognosis and response to immunotherapy of cancer patients [3, 4]. Also in vulvar high-grade squamous intraepithelial lesions (vHSIL), the precursor lesion of HPV^+^ VSCC, the prerequisite of a pre-existing well-coordinated hot immune microenvironment for response to different forms of immunotherapy has been identified [1, 5, 6].

In VSCC, the presence and impact of lymphoid cells on prognosis have been studied before [7] and revealed that the presence of high numbers of CD3^+^CD8^−^FOXP3^−^ T helper cells is an independent prognostic marker for survival, regardless of molecular subtype. Furthermore, higher T cell infiltration was associated with the expression of higher myeloid cell associated transcripts [8]. So far only two studies have analyzed myeloid cells in VSCC, reporting the association between high numbers of CD68^+^ macrophages and low-grade VSCC, and the presence of low CD1a^+^ dendritic cell numbers with recurrent VSCC [9, 10]. However, these studies were based on single marker immunohistochemistry in small patient cohorts; hence, no in-depth insight in the actual myeloid cell composition of VSCC is available.

Here, we aimed to fill that gap by an in-depth investigation of the myeloid and lymphoid infiltration in VSCC, using multispectral immunofluorescence and transcriptome analysis. Our study on a large sample set of healthy vulvar tissue and VSCC revealed that the type and number of VSCC infiltrating myeloid cells are linked to the molecular subtype of VSCC. High epithelial infiltration by CD14^+^CD68^−^CD11c^−^ monocytes formed a strong prognostic biomarker for excellent survival of VSCC patients, independent from CD3^+^ T cell infiltration.

## Materials and methods

### Patient samples

Formalin-fixed paraffin-embedded (FFPE) tissue of *n* = 104 women of ≥ 18 years old with histologically confirmed healthy HPV-negative vulva (*n* = 27) or VSCC (*n* = 77) was included. The healthy HPV^−^ vulvar tissue was from anonymized women who underwent labiaplasty. The included VSCC patients had FIGO stage I–IIIB at diagnosis, were treated with standard of care (surgical excision, and if needed adjuvant radiotherapy) at a tertiary hospital in the Netherlands, and all patients underwent 5 years of follow-up. All the tumor material investigated in this study was obtained prior to therapy. The study was in accordance with the Declaration of Helsinki and approved by the local medical ethical committee of the Leiden University Medical Center (LUMC) and in agreement with Dutch law. HPV, P16 and P53 typing on all formalin-fixed paraffin-embedded (FFPE) tumor sections was performed as described before [7]. HPV^+^p53mutant VSCCs were not observed in our cohort. Routine diagnostic beta-catenin immunohistochemical staining (BD, clone 14/beta-catenin) was performed on 10 VSCC patients. A summary of the patient characteristics, treatments and performed tumor analyses is provided in Supplemental Tables 1 and 2.

**Table 1:** Multivariate analyses

Covariate	Cox proportional HR (95% CI)	Adjusted *p*-value
Molecular subgroup (HPV^+^/HPV^−^p53wt/HPV^−^p53mut)	1.364 (0.515–3.613)	0.532
Stage (early/late)	2.120 (0.643–6.990)	0.217
Epithelium CD3^+^ total (low/high)	0.078 (0.009–0.638)	**0.017**
Epithelium CD14^+^rest^−^ (low/high)	0.077 (0.010–0.591)	**0.014**

Covariate	Cox proportional HR (95% CI)	Adjusted *p*-value
Molecular subgroup (HPV^+^/HPV^−^p53wt/HPV^−^p53mut)	1.317 (0.507–3.423)	0.572
Stage (early/late)	1.574 (0.475–5.213)	0.458
Epithelium CD3^+^ total (low/high)	0.112 (0.014–0.925)	**0.042**
Stroma HLADR^+^CD11c^+^CD14^+^rest^−^ (low/high)	0.121 (0.015–0.964)	**0.046**

Covariate	Cox proportional HR (95% CI)	Adjusted *p*-value
Molecular subgroup (HPV^+^/HPV^−^p53wt/HPV^−^p53mut)	1.177 (0.431–3.219)	0.750
Stage (early/late)	2.150 (0.651–7.096)	0.209
Epithelium CD3^+^ total (low/high)	0.079 (0.009–0.670)	**0.020**
Epithelium CD14^+^rest^−^ (low/high)	0.113 (0.014–0.939)	**0.044**
Stroma HLADR^+^CD11c^+^CD14^+^rest^−^ (low/high)	0.315 (0.037–2.704)	0.292

Bold values indicate the adjusted *p*-values that are < 0.05

### Multispectral immunofluorescence staining

A previously developed seven-color multispectral immunofluorescence panel for myeloid cells was applied, consisting of CD14, CD33, CD68, CD11c, CD163, HLADR and DAPI [6]. In this panel, a combination of direct detection (primary antibody directly labeled with fluorochrome) and indirect detection (fluorochrome labeled secondary antibody) of markers was used for fluorescence microscopy. An overview of the antibodies and staining methods included in each panel is shown in Supplemental Table 3. Briefly, 4-μm FFPE tissue sections were deparaffinized, endogenous peroxidase was blocked with hydrogen peroxide and heat induced epitope retrieval was performed with tris–EDTA (10 mM/1 mM, pH 9.0). SuperBlock (ThermoFisher Scientific) was used to block non-specific binding sites. First, the antibodies detected by Opal were applied, followed by the unconjugated antibodies which were incubated overnight. On the 2nd day, the corresponding fluorescently labeled secondary antibodies were applied, followed by 5 h incubation with the directly labeled primary antibodies. Finally, DAPI was applied as nuclear counterstain and slides were mounted [6].

### Quantification of immune cells in the TME

As published [5], images of the entire tissue sections stained with the multispectral immunofluorescence panels were acquired with the Vectra 3.0.5 multispectral imaging microscope (PerkinElmer) at 20× magnification. Immune cells in the TME were automatically phenotyped and counted with inForm 2.4 image analysis software (PerkinElmer-Akoya Biosciences) after manual training. The software was trained to segment epithelium and stroma, segment DAPI^+^ nucleated cells and assign a phenotype to each cell. All phenotypes were visually inspected on accurateness, and if errors were detected, the training was further optimized until all discrepancies were resolved. Immune cell counts were normalized for tissue size (cells/mm^2^ epithelium and cells/mm^2^ stroma). A threshold of a median cell count ≥ 10 cells/mm^2^ in at least one tissue category was applied to study biologically common phenotypes.

### Transcriptomic analyses

The archived diagnostic FFPE tissue blocks were used to cut sections of 10 μm for RNA extraction [8]. Directly before and after these 10-μm-thick sections, a 4-μm section was cut for hematoxylin–eosin (HE) staining. A pathologist annotated the tumor regions on the HE slides to ensure that the slides contained tumor. After the sections were deparaffinized, the tumor areas were macro-dissected using the annotated HE slides as reference. RNA was then isolated with the RNA FFPE isolation kit (Qiagen), according to the manufacturer’s instructions. The quality and quantity of the RNA were verified with the Agilent 2100 Bioanalyzer System, using the RNA 6000 Nano kit (Agilent). Samples were selected for further processing when > 20% of the RNA fragments were > 300 bp and the corrected RNA concentration remained ≥ 60 μg/µl.

To measure the gene expression profiles, 300 ng of RNA from each sample was hybridized with the probes of the human PanCancer panel (730 genes) or IO360 panel (750 genes) for 17 h at 65 °C, following the manufacturer’s instructions (NanoString). The number of copies of each gene in every sample was counted by scanning 490 Fields Of View using the NanoString nCounter MAX system. The raw data (counts of genes) were uploaded to the Rosalind platform, after which quality checks were performed. The housekeeping genes in the panels facilitated sample-to-sample normalization. Eight negative controls and six synthetic positive controls were included. The data were reviewed for reliability and validity based on the imaging (> 0.75) and binding density (0.1–2.25) quality control metrics and performance check of the positive controls (0.95–1). Benjamini–Hochberg-adjusted *p*-values were used to decrease the false-discovery rate. Differentially expressed genes (DEGs) were defined by a log2 fold difference of > 1 or ≤ 1 and a *p*-value < 0.05.

Gene ontology analysis of the differentially expressed genes (DEGs) was performed with ClueGo [11].

Gene set enrichment analysis (GSEA) was performed with the Broad Institute GSEA software package and collections for human gene sets, version 4.3.2. [12, 13] One thousand permutations were applied, and small gene sets were excluded by applying a threshold of at least 15 genes. Gene sets with an FDR < 0.25 as well as *p*-value < 0.05 were selected.

### Statistical analyses

Statistical data analysis was performed with GraphPad Prism 9.3.1, which was also used to create graphs to visualize the data. The median immune counts of three different VSCC molecular subtypes were compared with the non-parametric Kruskal–Wallis test, of two subgroups with the non-parametric Mann–Whitney U test. Spearman correlation was used to study correlations between the T cell and myeloid cell infiltrate. Kaplan–Meier survival analyses were performed with SPSS 25.0. Differences in survival and hazard ratio (HR) with 95% confidence interval were calculated with the non-parametric Mantel-Cox test. Two-sided *p*-values < 0.05 were marked as statistically significant.

## Results

### A diverse myeloid cell infiltration is present in VSCC

**Fig. 1:**
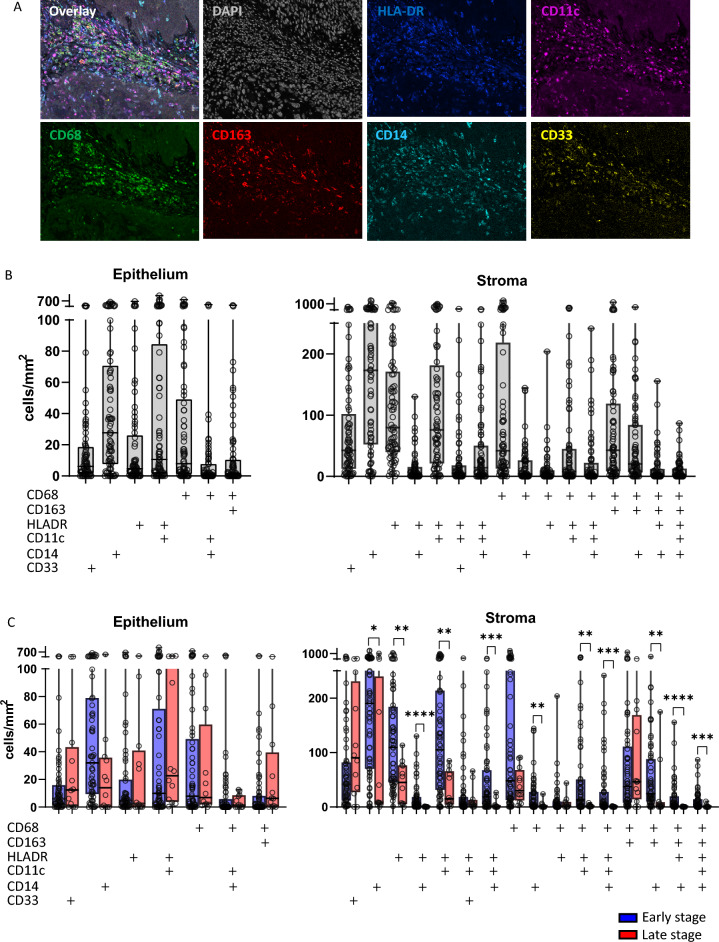
Identification and quantification of myeloid cells in VSCC. **A** Multispectral immunofluorescence staining of myeloid cells in VSCC, single markers staining and overlay. **B** Myeloid cell phenotypes and quantification in the total VSCC cohort (*n* = 77). **C** Myeloid cell phenotypes and quantification in early (FIGO I/II in blue, *n* = 65) versus late (FIGO III in red, *n* = 12) stage VSCC. Boxplots with middle line indicating median, colored box indicating interquartile range, vertical lines from minimum to maximum cell count value. Marker + indicates its expression by that specific cell phenotype. Asterisks indicate statistically significant differences (*p < 0.05, **p < 0.01, ***p < 0.001, ****p < 0.0001)

An earlier transcriptomic study suggested that stronger T cell infiltration in VSCC was associated with more myeloid cell infiltration [8], but the actual composition and location of the myeloid cell infiltrate were unknown. Therefore, a multispectral myeloid cell immunofluorescence panel, recently shown to be helpful in understanding the clinical response of vHSIL patients to different forms of immunotherapy [5, 6], was applied to a large set of 77 VSCC patients (Fig. 1A).

Based on the markers CD68, CD163, HLADR, CD11c, CD14 and CD33 used, at least 16 different phenotypes of myeloid cells that infiltrated VSCC were identified. To simplify their notation, the positively stained markers are indicated with a ‘ + ’, whereas markers that were negative are indicated as ‘rest-’. The presence of CD14^+^rest^−^ monocytes, CD68^+^rest^−^ macrophages and HLADR^+^CD11c^+^rest^−^ dendritic cells was most prominent in the epithelium. The stroma of VSCC contained most of the phenotypes and was richly infiltrated by the three afore-mentioned myeloid cell subtypes. In addition, CD68^+^rest^−^ macrophages and CD68^+^CD163^+^rest^−^ M2-like macrophages were detected (Fig. 1B). However, there was a large diversity regarding the numbers of tumor-infiltrating myeloid cells across the 77 patients.

Subgroup analysis based on early stage (FIGO I/II) versus late stage (FIGO III) VSCC showed no differences in epithelial myeloid cell infiltration. However, in the stromal compartment of late stage VSCC, a significant decrease in CD14^+^CD33^−^rest^−^ monocytes, HLADR^+^rest^−^ cells, HLADR^+^ CD14^+^rest^−^ monocytes, HLADR^+^CD11c^+^rest^−^ dendritic cells, HLADR^+^CD11c^+^CD14^+^rest^−^ dendritic cells (DCs), CD68^+^ CD14^+^rest^−^ inflammatory macrophages, CD68^+^ HLADR^+^CD11c^+^rest^−^ cells, CD68^+^ HLADR^+^CD11c^+^CD14^+^rest^−^ M1-like macrophages, CD68^+^CD163^+^HLADR^+/− −^CD14^+^rest^−^ M2-like macrophages and CD68^+^CD163^+^HLADR^+^CD11c^+^CD14^+^CD33^−^ M2-like macrophages was observed (Fig. 1C). This indicates that during progression of VSCC from early to late stage, the tumor may become colder in terms of myeloid cell infiltration (Supplemental Table 4).

### Molecular subtype specific myeloid cell attraction in vulvar cancer

**Fig. 2:**
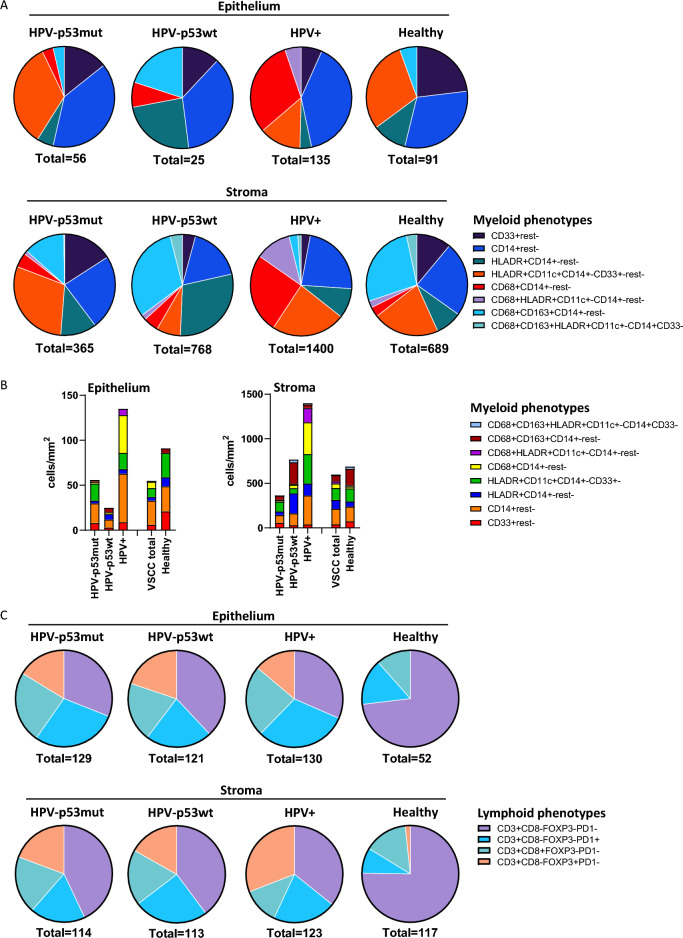
Myeloid and lymphoid immune composition differences per VSCC molecular subtype. The myeloid cell composition **A** fractionally and **B** quantitatively, as well as **C** the lymphoid cell composition fractionally in the epithelium and stroma of HPV^−^p53mut VSCC (*n* = 31), HPV^−^p53wt VSCC (*n* = 21), HPV^+^ VSCC (*n* = 25) and healthy HPV^−^ vulva (*n* = 10). Number below the graphs indicates the median total number of cells/mm.^2^. Cell types are indicated by the markers (CD68, CD163, HLADR, CD11c, CD14, and CD33) staining positive, markers not stained are indicated as ‘Rest-’

VSCC can be subdivided into three different molecular subtypes which are correlated to survival, based on the presence of HPV and the TP53 status: HPV^−^p53mutant, HPV^−^p53wildtype and HPV^+^. To assess if the increased myeloid cell infiltrate in VSCC was a general phenomenon or specific for a molecular subtype, the myeloid cell numbers and composition in each subgroup were studied. HPV^+^ VSCC had a higher number of total infiltrating myeloid cells (both intraepithelial and stromal), with especially more CD14^+^rest^−^ monocytes, HLADR^+^CD11c^+^CD14^+^rest^−^ inflammatory DCs and CD68^+^ rest^−^ macrophages, but less CD68^+^CD163^+^rest^−^ M2-like macrophages when compared to the HPV^−^ subtypes. HPV^−^p53wt VSCC had more stromal HLADR^+^ CD14^+−^rest^−^ cells, HLADR^+^CD11c^+^CD14^+−^CD33^+^ dendritic cells, CD68^+^ HLADR^+−^ CD14^+−^rest^−^ M1-like macrophages and CD68^+^CD163^+^HLADR^+−^ CD14^+−^rest^−^ M2-like macrophages compared to HPV^−^p53mut VSCC (Fig. 2A and B, Supplemental Table 5A, Supplemental Fig. 1). Twenty out of 23 myeloid subpopulations were statistically significantly different between the VSCC molecular subtypes. In contrast, re-analysis of the number and composition of detected subpopulations of infiltrating T cells [7] showed that they did not grossly differ across the molecular subtypes of VSCC (Fig. 2C, Supplemental Table 5B, Supplemental Figs. 2 and 3A).

Since coordination of immunity has been a well-established prognosticator for survival and response to immunotherapy [3, 6, 14], we analyzed the correlations between the lymphoid and myeloid cell infiltrate. No clear correlations were found between lymphoid and myeloid cell infiltration in all VSCC molecular subtypes, except for a weak but significant correlation between stromal CD3^+^PD1^+^ T cells and epithelial CD14^+^rest^−^ myeloid cells in HPV^+^ VSCC (Supplemental Fig. 4).

### Key myeloid cell phenotypes impacting survival in VSCC

**Fig. 3:**
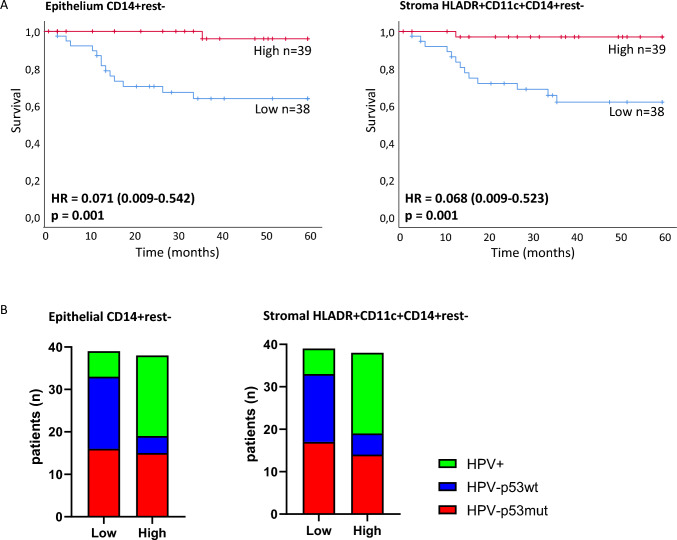
Pro-inflammatory myeloid cell composition predicts excellent survival in VSCC. **A** Kaplan–Meier survival curves for VSCC (*n* = 77) with high or low myeloid cell infiltration, including hazard ratio (HR, between brackets the 95% confidence interval of the hazard ratio is provided), and *p*-value. Total VSCC cohort split by median into high versus low counts. Left survival curve for separation by CD14^+^rest^−^ monocyte infiltration, low infiltration (blue): HPV^−^p53mut *n* = 16, HPV^−^p53wt *n* = 17, HPV^+^ *n* = 6, high infiltration (red): HPV^−^p53mut *n* = 15, HPV^−^p53wt *n* = 4, HPV^+^ *n* = 19. Right survival curve for separation by stromal HLADR^+^CD11c^+^CD14^+^rest^−^ dendritic cell infiltration, low infiltration (blue): HPV^−^p53mut *n* = 17, HPV^−^p53wt *n* = 16, HPV^+^ *n* = 6, high infiltration (red): HPV^−^p53mut *n* = 14, HPV^−^p53wt *n* = 5, HPV^+^ *n* = 19. **B** Distribution of VSCC molecular subtypes (*n* = 77 total) across low versus high (split by median) epithelial CD14^+^rest^−^ monocyte infiltration (left graph), and stromal HLADR^+^CD11c^+^CD14^+^rest.^−^ dendritic cell infiltration (right graph)

We performed Kaplan–Meier survival analyses using the median counts of each myeloid cell phenotype to split the total VSCC cohort into high or low infiltrated. This revealed three myeloid cell phenotypes that strongly correlated with survival (Fig. 3A, Supplemental Fig. 5). The strongest separation of the survival curves was observed for epithelial CD14^+^rest^−^ monocytes and stromal CD14^+^CD11c^+^HLADR^+^rest^−^ inflammatory DCs (both *p*-value = 0.001) (Fig. 3A). Patients from all three molecular subtypes were represented in these myeloid cell high and low groups (Fig. 3B). Intriguingly, patients with VSCC in which the function of p53 (in)directly is impaired (HPV^+^ or HPV^−^p53mut) more often showed high infiltration with epithelial CD14^+^rest^−^ monocytes and stromal CD14^+^CD11c^+^HLADR^+^rest^−^rest^−^ inflammatory DCs, compared to HPV^−^p53wt VSCC (Fig. 3B). Functional loss of p53 in cancer cells has previously been linked to enhanced recruitment of myeloid cells to the tumor site [15–17].

### Transcriptomic differences in myeloid hot versus cold tumors

**Fig. 4:**
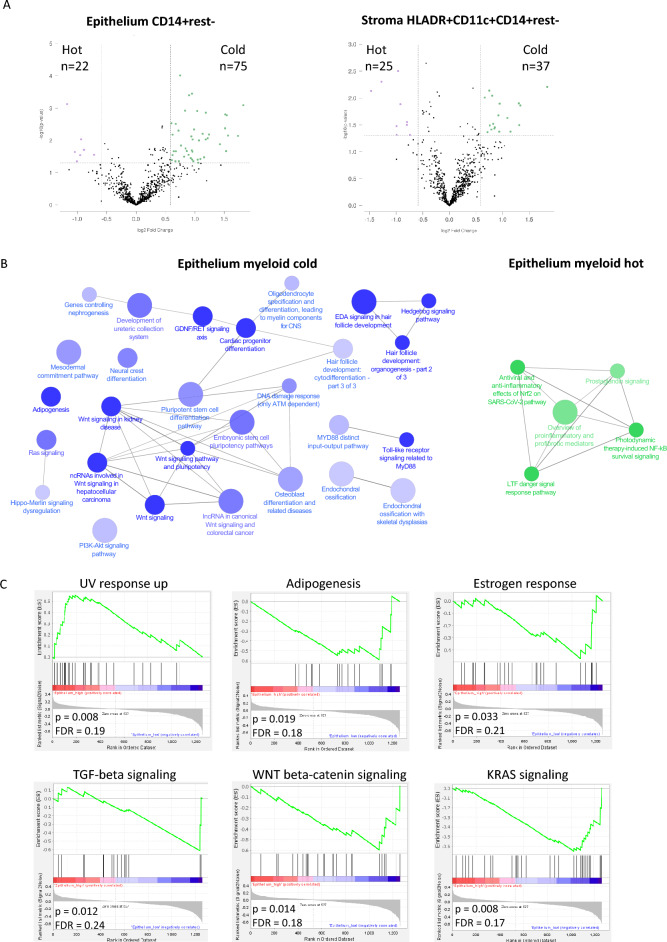
Transcriptomic differences between myeloid hot and cold tumors. **A** Volcano plots of differentially expressed genes (DEGs) between myeloid hot versus cold VSCC lesions (total VSCC *n* = 77, split by median into hot or cold): epithelial CD14^+^rest^−^ monocytes (left graph) and stromal HLADR^+^CD11c^+^CD14^+^rest^−^ dendritic cells (right graph). **B** ClueGo pathway analysis of identified DEGs between epithelial CD14^+^rest^−^ monocytes hot versus cold VSCC. **C** Hallmark gene set enrichment analysis (GSEA), showing gene sets enriched in epithelial CD14^+^rest.^−^ hot versus cold VSCC, selected based on FDR < 0.25 as well as *p* < 0.05

In order to gain more insight into what may underly the difference between specific myeloid cell phenotype hot and cold tumors, we combined the transcriptomic data (1258 different individual genes) of a group of 44 VSCC patients, containing both early and late stage patients, and all three molecular subtypes [8]. These patients were divided in two groups based on the median epithelial CD14^+^rest^−^ monocyte cell count or the stromal CD14^+^CD11c^+^HLADR^+^rest^−^ inflammatory DC count, and the differentially expressed genes (DEGs) between the myeloid hot versus cold groups were analyzed. This rendered a total of 97 DEGs between the epithelial CD14^+^rest^−^ monocyte high versus low group (Fig. 4A, Supplemental Table 6), and a total of 62 DEGs between the stromal inflammatory DC high versus low group (Fig. 4A, Supplemental Table 7), using a threshold of fold change difference > 1.5 and *p*-value < 0.05. 29 DEGs were present in both comparisons. The great majority of DEGs were higher expressed in myeloid cold tumors (75 and 37, respectively).

In contrast to the stromal region, the epithelial region is unambiguously demarcated and as such, more reproducible to define across studies. Therefore, we focused on the epithelial CD14^+^rest^−^ monocytes high/low separation in subsequent analyses. The DEGs between CD14^+^rest^−^ monocyte high and low VSCCs comprised a number of cytokines, including increased expression of *CXCL8* and *CCL20,* known to attract monocytes [18, 19], as well as the inducers of inflammation *IL-1α* and *IL-1β* (Supplemental Table 6)*,* previously reported to be upregulated in tumors that have lost p53 function [15]*.* The cytokines *IL-34, CCL14* and *CCL19* that were overexpressed in the CD14^+^rest^−^ monocyte low VSCC (Supplemental Table 6) are not known to recruit monocytes. Analysis of the differentially expressed cytokines revealed no overt differences between the VSCC molecular subtypes (Supplemental Fig. 6).

To analyze which underlying signaling pathways were activated in these myeloid hot and cold tumors, ClueGo gene ontology analysis and Hallmark Gene Set Enrichment Analysis (GSEA) were performed. The majority of upregulated genes in CD14^+^rest^−^ monocyte cold tumors were involved in organogenesis pathways, including ‘pluripotent stem cell differentiation pathway’, ‘embryonic stem cell pluripotency pathway’, ‘WNT signaling pathway and pluripotency’. In CD14^+^rest^−^ monocyte hot tumors, the majority of upregulated genes were involved in pro-inflammatory pathways (Fig. 4B, Supplemental Fig. 7). The GSEA revealed that epithelial monocyte hot VSCCs displayed a gene set upregulated in the response to UV, known to lead to monocyte attraction [20]. In contrast, epithelial monocyte cold VSCCs were characterized by the upregulation of genes involved in adipogenesis and in the oncogenic KRAS, WNT and TGFβ signaling pathways (Fig. 4C). However, the upregulation of the WNT pathway in monocyte cold VSCC could not be confirmed at the protein level by beta-catenin immunohistochemistry (Supplemental Fig. 8).

### Myeloid and T cell counts are independent predictors for excellent survival in VSCC

**Fig. 5:**
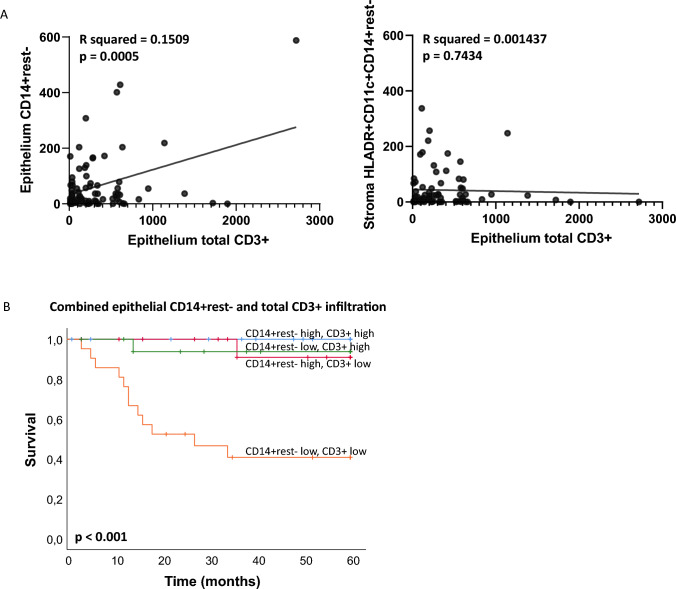
High myeloid cell and T cell infiltration are independent predictors for excellent survival. **A** Pearson correlation plots between myeloid (epithelial CD14^+^rest^−^ or stromal HLADR^+^CD11c^+^CD14^+^rest^−^) and lymphoid cell infiltrate (epithelial total CD3^+^) in VSCC (*n* = 77). **B** Kaplan–Meier survival curves for VSCC patients with differential lymphoid (epithelial total CD3^+^) and myeloid (epithelial CD14^+^rest.^−^) cell infiltration (total VSCC *n* = 77, split by respective medians into hot or cold)

Previously, it was described that HPV^−^p53mut VSCC patients have the poorest survival, HPV^−^p53wt VSCC patients an intermediate survival and HPV^+^ VSCC patients an excellent survival [2], similar to our study cohort (Supplemental Fig. 9). In addition, epithelial CD3^+^ T cell infiltration was reported to be prognostic in VSCC, irrespective of tumor stage and molecular subtype [7, 8]. As there was no direct correlation between epithelial T cell infiltrate and either identified myeloid cell subpopulation (Fig. 5A), we explored whether the combination of intraepithelial T cell infiltration with both myeloid cell subpopulations would provide more insight into the prognostic role of either.

Multivariate analyses with tumor stage, molecular subtype, intraepithelial T cell infiltration and either one of the myeloid populations (epithelial monocytes and stromal inflammatory DCs) showed that both myeloid cell types could function as prognostic factors (Table 1). However, analysis of all three immune markers simultaneously revealed that both intraepithelial CD3^+^ T cells and the intraepithelial CD14^+^rest^−^ monocytes were the only independent prognostic markers, irrespective of tumor stage and molecular subtype (Table 1). An interaction analysis of both latter phenotypes, by splitting the patient group into four based on the median epithelium infiltrating CD3^+^ T cell count (high/low) [7] and the median epithelium infiltrating CD14^+^rest^−^ monocyte count, revealed that the group of patients with a combined high CD3^+^ T cell and high CD14^+^rest^−^ monocyte infiltration displayed the best survival, similar to the patient groups with a high infiltration of either one of these two immune cell types. The patient group with a low infiltration of both immune cell types displayed poorest survival (Fig. 5B). This revealed that the presence of a high epithelial CD14^+^rest^−^ monocyte infiltrate is equally important to that of T cells.

## Discussion

The data from this first in-depth study of myeloid cell infiltration in a large group of VSCCs revealed that the composition of the myeloid cell infiltrate strongly differed per molecular subtype of VSCC, while this is not the case for lymphoid cell infiltrate [7]. Among the 16 different identified phenotypes, two myeloid cell phenotypes were strongly associated with improved survival: epithelial monocytes and stromal dendritic cells. These myeloid cell subsets were found in higher numbers more often in VSCCs with loss of p53 function, either through interaction with the HPV oncoprotein E6 or via direct TP53 mutation. In comparison with all other known prognostic parameters (tumor stage, molecular subtype and intraepithelial T cell infiltration), only intraepithelial monocytes and T cells were independent prognostic factors in a multivariate analysis. A strong epithelial infiltration with either T cells or monocytes was associated with drastically improved clinical benefit, while low infiltration with both immune cell types was associated with poor survival.

It is well-established that molecular features of the tumor may impact the immune composition of the TME [15, 21–26]. While our earlier studies in VSCC revealed no overt impact of the different molecular subtypes on the number and composition of lymphocyte infiltration [7, 9], our current study suggests that this is different for myeloid cells. There were clear differences with respect to the numbers and phenotypes of infiltrating myeloid cells between molecular subtypes. In particular patients with VSCC not caused by HPV or a TP53 mutation, they were less likely to show strong infiltration with intraepithelial monocytes and stromal DCs and immune cell types both shown to be strongly associated with patient survival. Earlier studies have shown that loss of p53 function specifically resulted in the production of cytokines and attraction of innate leukocytes, the specific cytokine and innate cell types depending on the mouse models used [17, 24] and included monocytes [16]. Our data suggest that in VSCC, functional loss of p53 may lead to the enhanced attraction of intraepithelial monocytes and stromal DCs.

At first sight, it is surprising to find that the epithelial infiltration of VSCC with monocytes forms such a strong independent prognostic factor. However, monocytes attracted by CCL20 were shown to be essential for efficient cross-priming of T cells after mucosal or skin immunization and preceded the sequential accumulation of CD11c^+^ MHC class II^+^ DCs in dermis and epithelium [19]. While there is evidence that monocytes may present the antigen directly to T cells [19], others have found that they were highly effective in transferring antigens to DCs in lymphoid tissue [27], in line with current concepts of antigen cross-priming [28]. Also in VSCC, the increased presence of monocytes is associated with increased expression of CCL20 in tumors, making it a highly likely scenario that in VSCC, monocytes may have a similar role. Interestingly, in esophageal cancer, high ‘tumor monocyte content’ was recently found to be a strong predictor for response to immunotherapy and long survival, outperforming tumor lymphocyte content [29].

In conclusion, a dense myeloid and/or lymphoid infiltrate predicts excellent survival in VSCC patients. A hot tumor immune microenvironment generally is associated with better clinical outcome and improved response to different forms of immunotherapy [4, 30, 31]. Although many patients with late stage VSCC displayed infiltration by multiple immune cell types, overall, this group of patients showed less infiltrating myeloid cells and lymphocytes [7, 9] than patients with early stage VSCC. For the clinical application of immunotherapy, this suggests that treatment should be provided as early as possible. Based on these findings, a clinical trial exploring the potential of neoadjuvant checkpoint blockade in primary VSCC has started this year (NCT05761132).

## Supplementary Information

Below is the link to the electronic supplementary material.

Supplementary file1 (PDF 21173 KB)

Supplementary file2 (XLSX 44 KB)

## Data Availability

All data generated during this study are included in this paper and are available upon reasonable request.
