# Supplementary material for: Monocyte infiltration is an independent positive prognostic biomarker in vulvar squamous cell carcinoma
Source: Cancer Immunol Immunother. 2024 Jul 2;73(9):166. doi: 10.1007/s00262-024-03755-w (PMC11219697; doi:10.1007/s00262-024-03755-w)
Supplement: Supplementary file 1 — Supplementary file1 (PDF 21173 KB) [file 262_2024_3755_MOESM1_ESM.pdf]

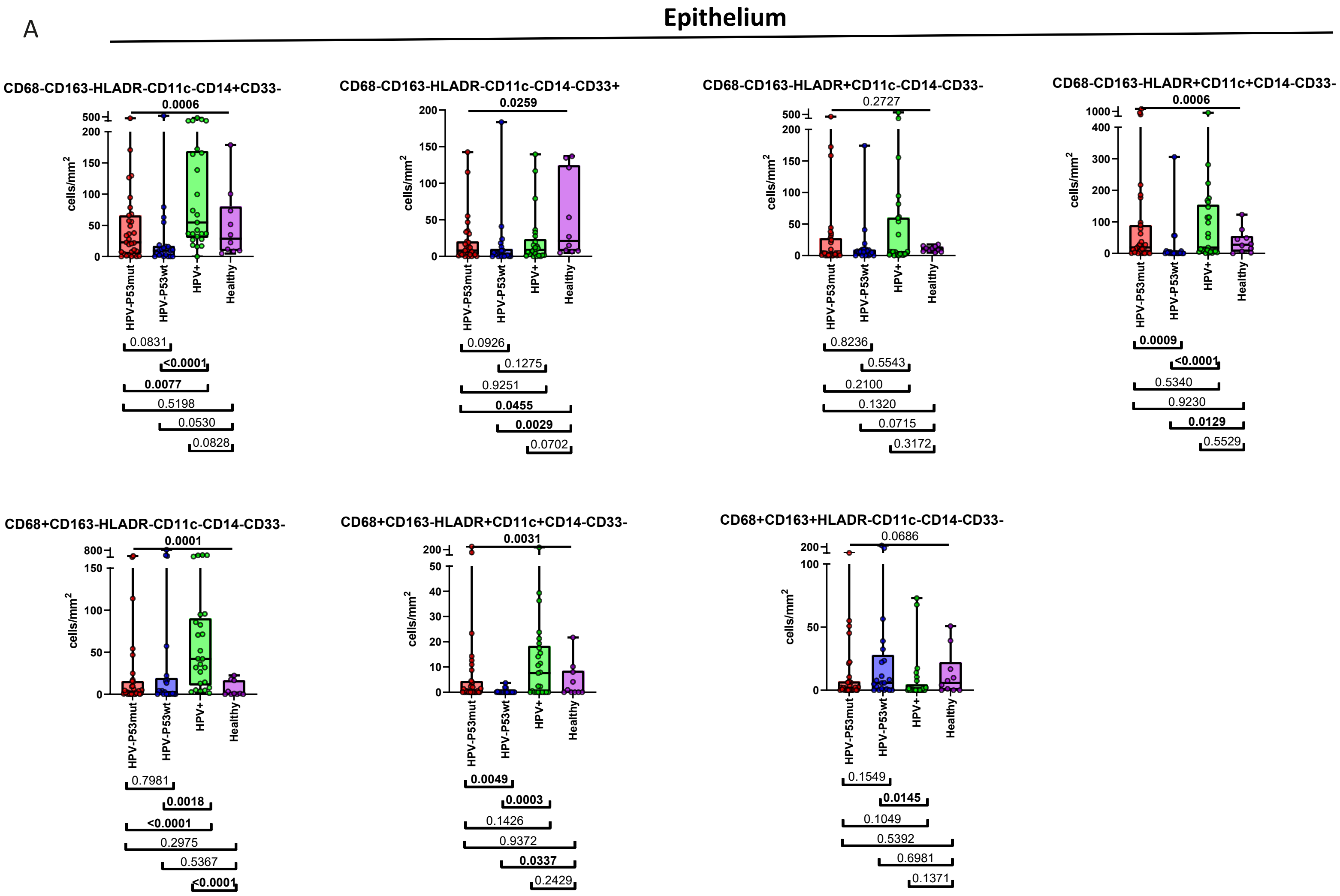

Supplemental Figure 1. Myeloid infiltrate per VSCC molecular subgroup and in healthy vulva.
A) Myeloid infiltrate in the epithelium. HPV-P53mut VSCC n=31, HPV-P53wt VSCC n=21, HPV+ VSCC n=25, healthy HPV- vulva n=10. Boxplots with middle line indicating median, colored box indicating interquartile range, vertical lines from minimum to maximum. Kruskal Wallis test derived p-value depicted on top of the boxplots, indicating a significant difference across the four groups. Mann-Whitney U test derived p-values depicted below the boxplots, indicating a significant difference between two groups.

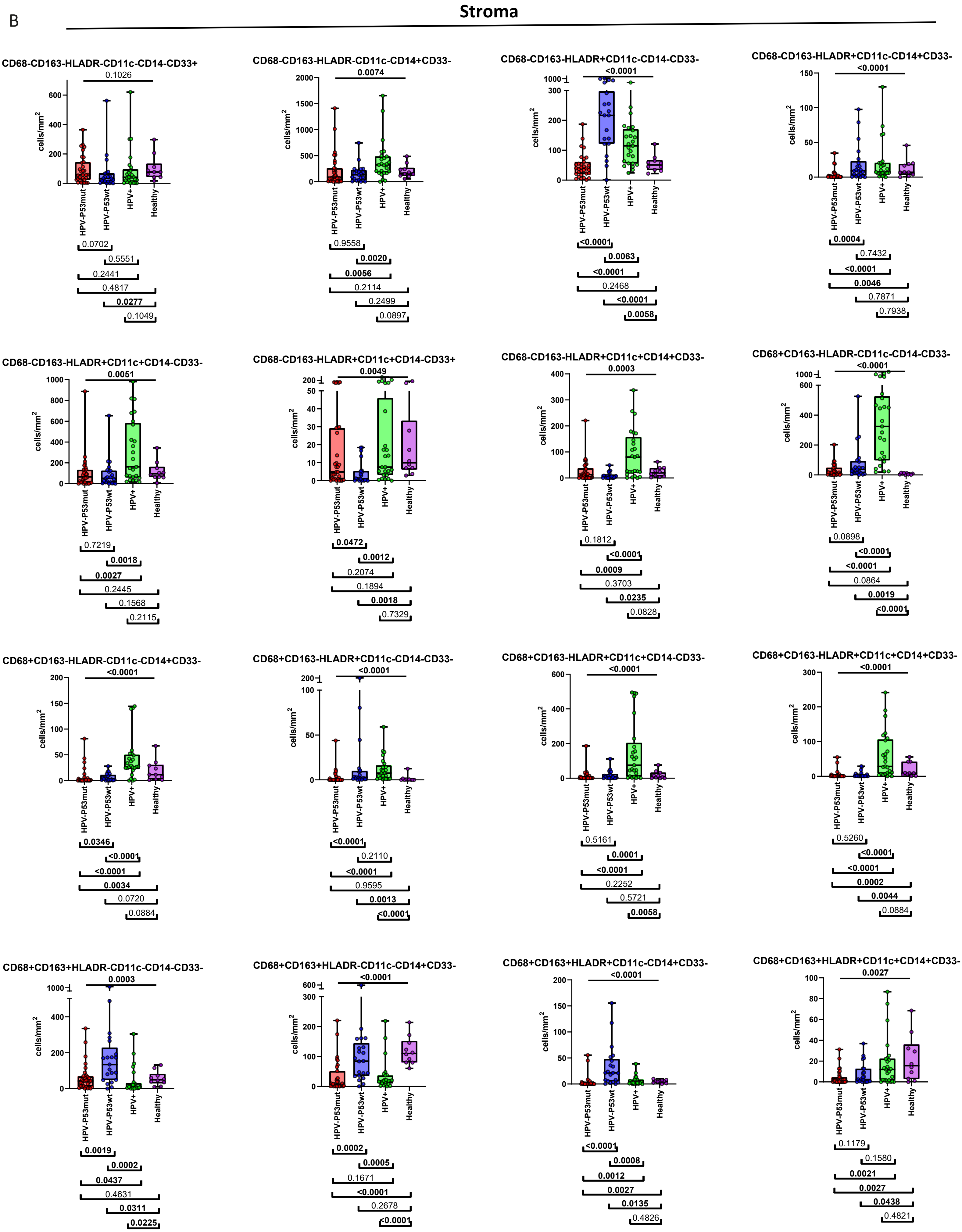

Supplemental Figure 1. Myeloid infiltrate per VSCC molecular subgroup and in healthy vulva.
B) Myeloid infiltrate in the stroma. HPV-P53mut VSCC n=31, HPV-P53wt VSCC n=21, HPV+ VSCC n=25, healthy HPV- vulva n=10. Boxplots with middle line indicating median, colored box indicating interquartile range, vertical lines from minimum to maximum. Kruskal Wallis test derived p-value depicted on top of the boxplots, indicating a significant difference across the four groups. Mann-Whitney U test derived p-values depicted below the boxplots, indicating a significant difference between two groups.

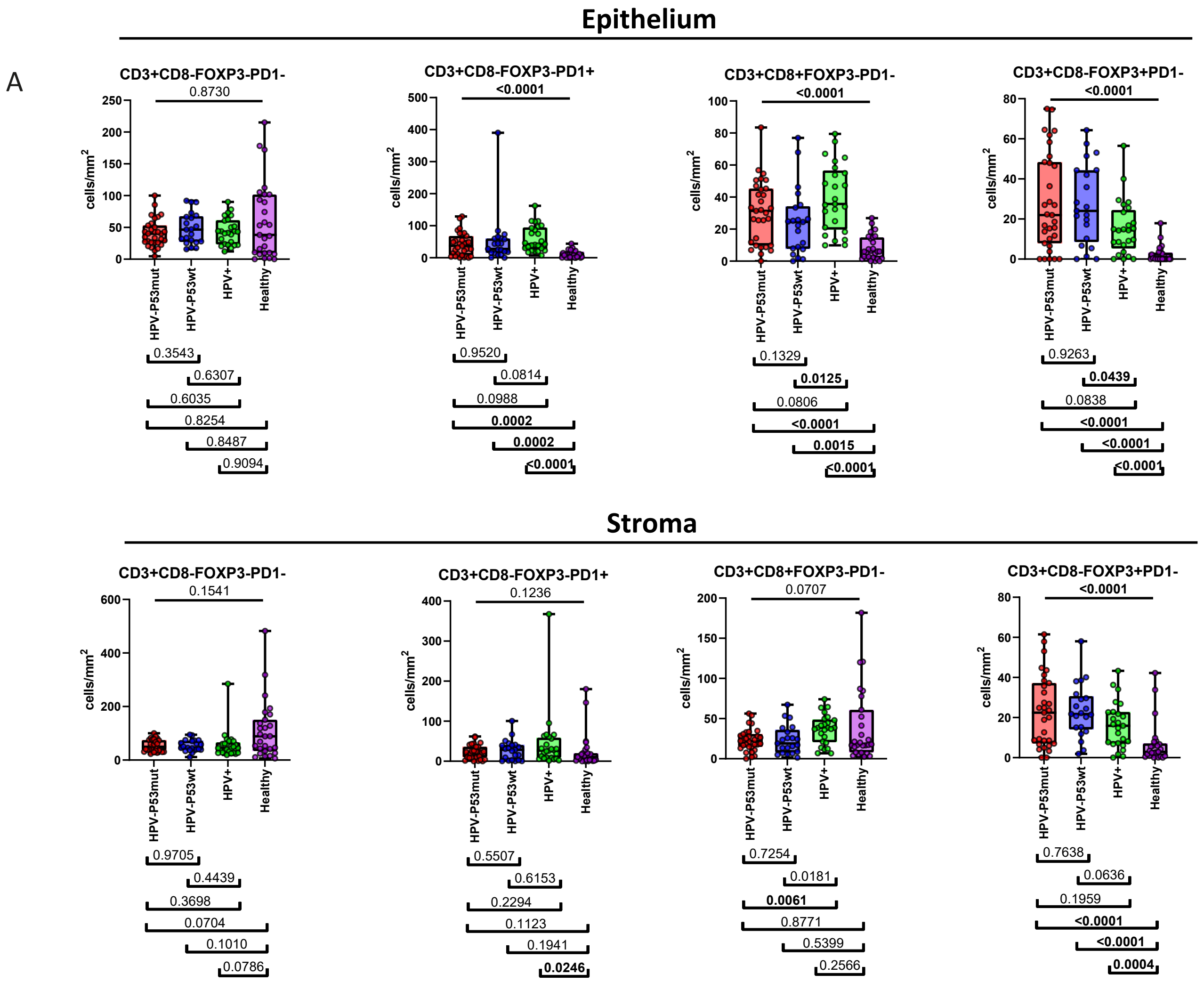

Supplemental Figure 2. Lymphoid infiltrate per VSCC molecular subgroup and in healthy vulva.
A) Lymphoid infiltrate in the epithelium. B) lymphoid infiltrate in the stroma.
HPV-P53mut VSCC n=31, HPV-P53wt VSCC n=21, HPV+ VSCC n=25, healthy HPV- vulva n=27.
Boxplots with middle line indicating median, colored box indicating interquartile range, vertical lines from minimum to maximum.
Kruskal Wallis test dervied p-value depictend on top of the boxplots, indicating a significant dfiference across the four groups.
Mann-Whitney U test derived p-values depicted below the boxplots, indicating a significant difference between two groups.

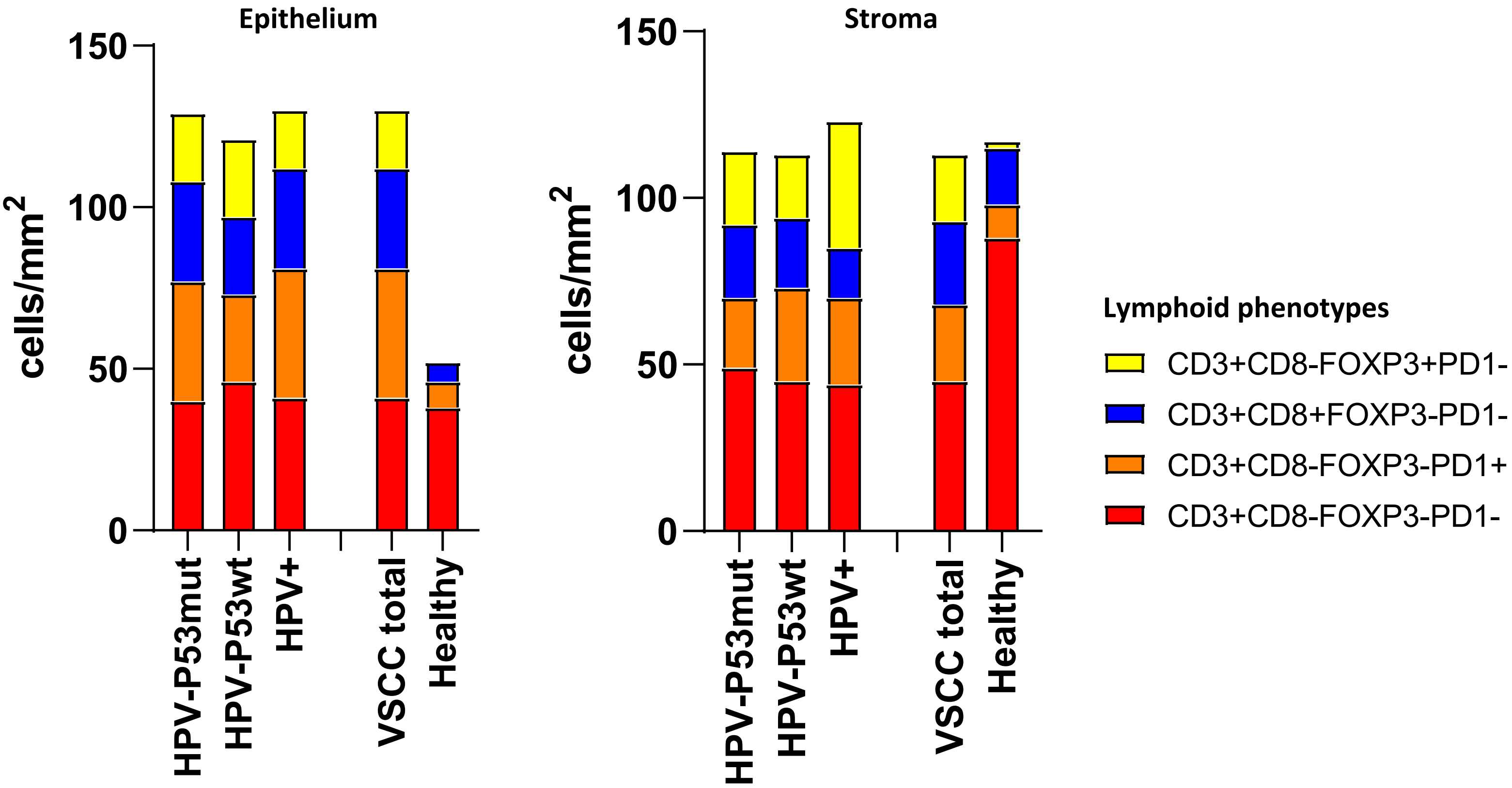

Supplemental Figure 3. No lymphoid immune composition differences across VSCC molecular subgroups. HPV-P53mut VSCC n=31, HPV-P53wt VSCC n=21, HPV+ VSCC n=25, healthy HPV- vulva n=10.

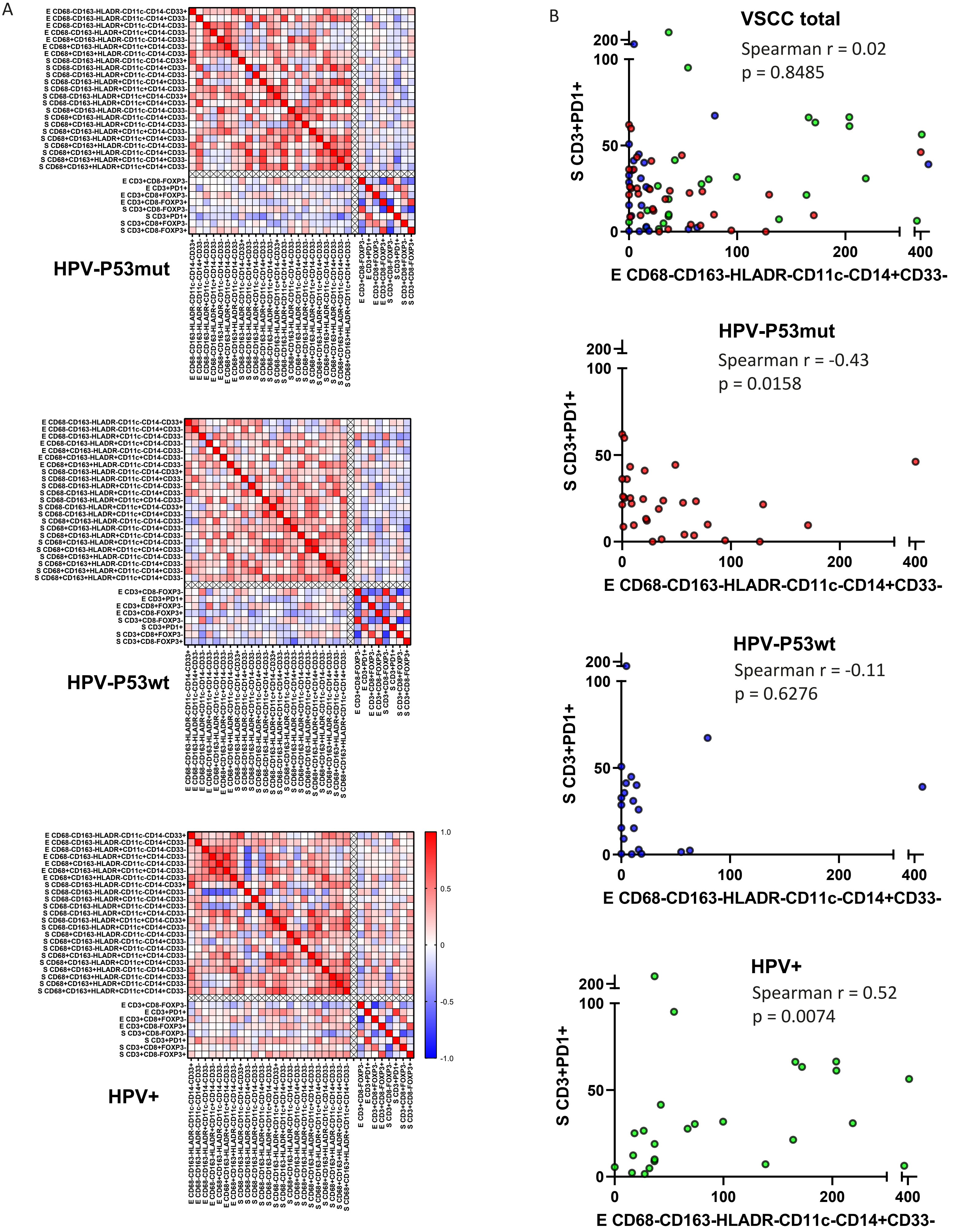

Supplemental Figure 4. Correlation of myeloid and lymphoid immune infiltrate per VSCC molecular subgroup.
A) Spearman correlation heatmap between myeloid and lymphoid immune phenotypes, per VSCC molecular subgroup. HPV-P53mut VSCC n=31, HPV-P53wt VSCC n=21, HPV+ VSCC n=25. B) Correlation dot plots between stromal CD3+PD1+ T cells and epithelial CD68-HLADR-CD11c-CD14+CD33- monocytes. Visualized for the total VSCC cohort n=77, and per molecular subgroup separately: HPV-P53mut VSCC (red) n=31, HPV-P53wt VSCC (blue) n=21, HPV+ VSCC (green) n=25.

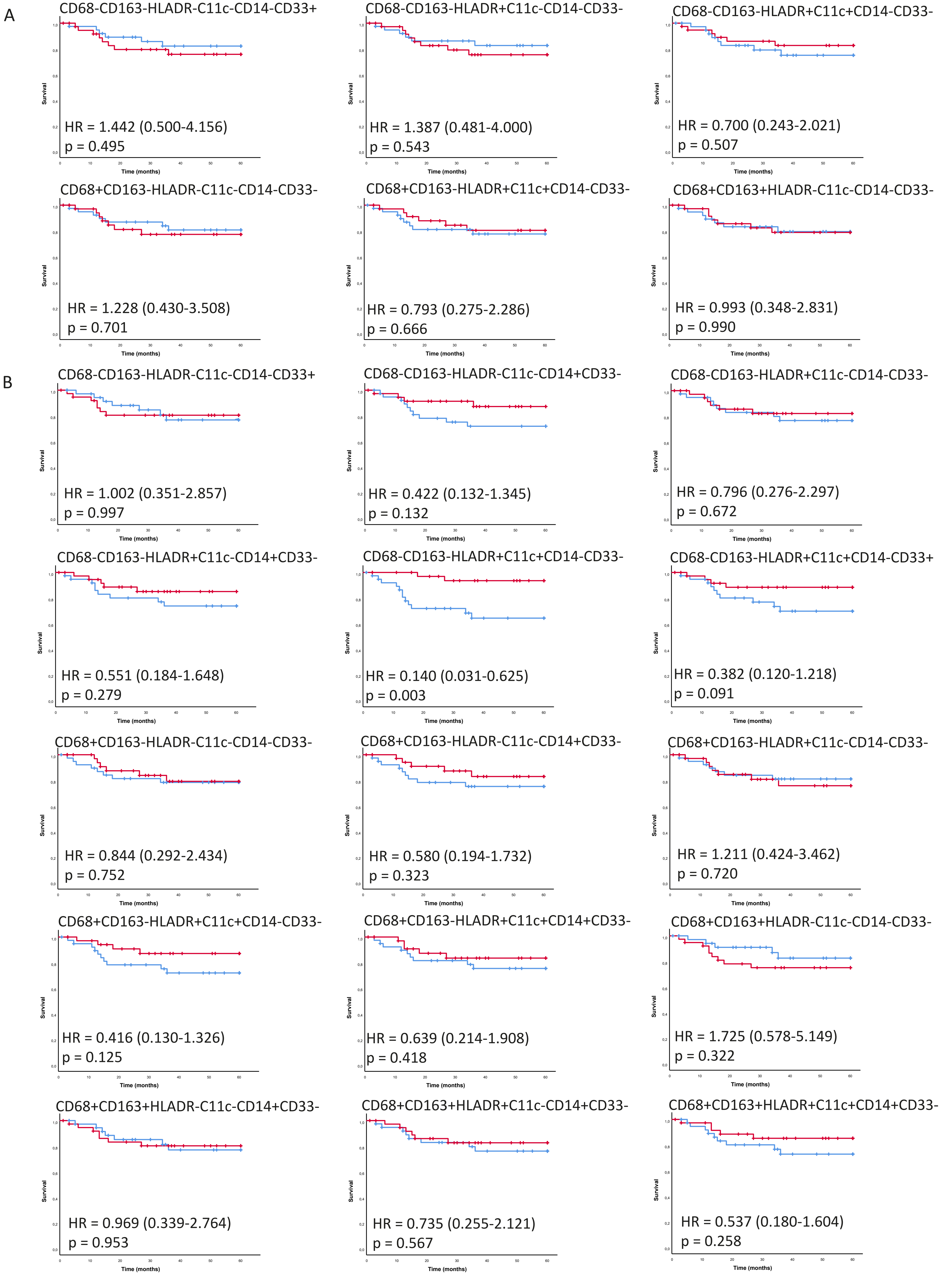

Supplemental Figure 5. Impact of myeloid cell phenotypes presence on survival of VSCC patients.
The median counts of each myeloid cell phenotype were used to split the total VSCC cohort (n=77) into high (red) or low (blue) infiltrated. The respective Kaplan Meier curves are depicted in A) for the epithelial phenotypes and in B) for the stromal phenotypes, including the hazard ratio (HR, between brackets the 95% confidence interval of the hazard ratio is provided) and p-value.

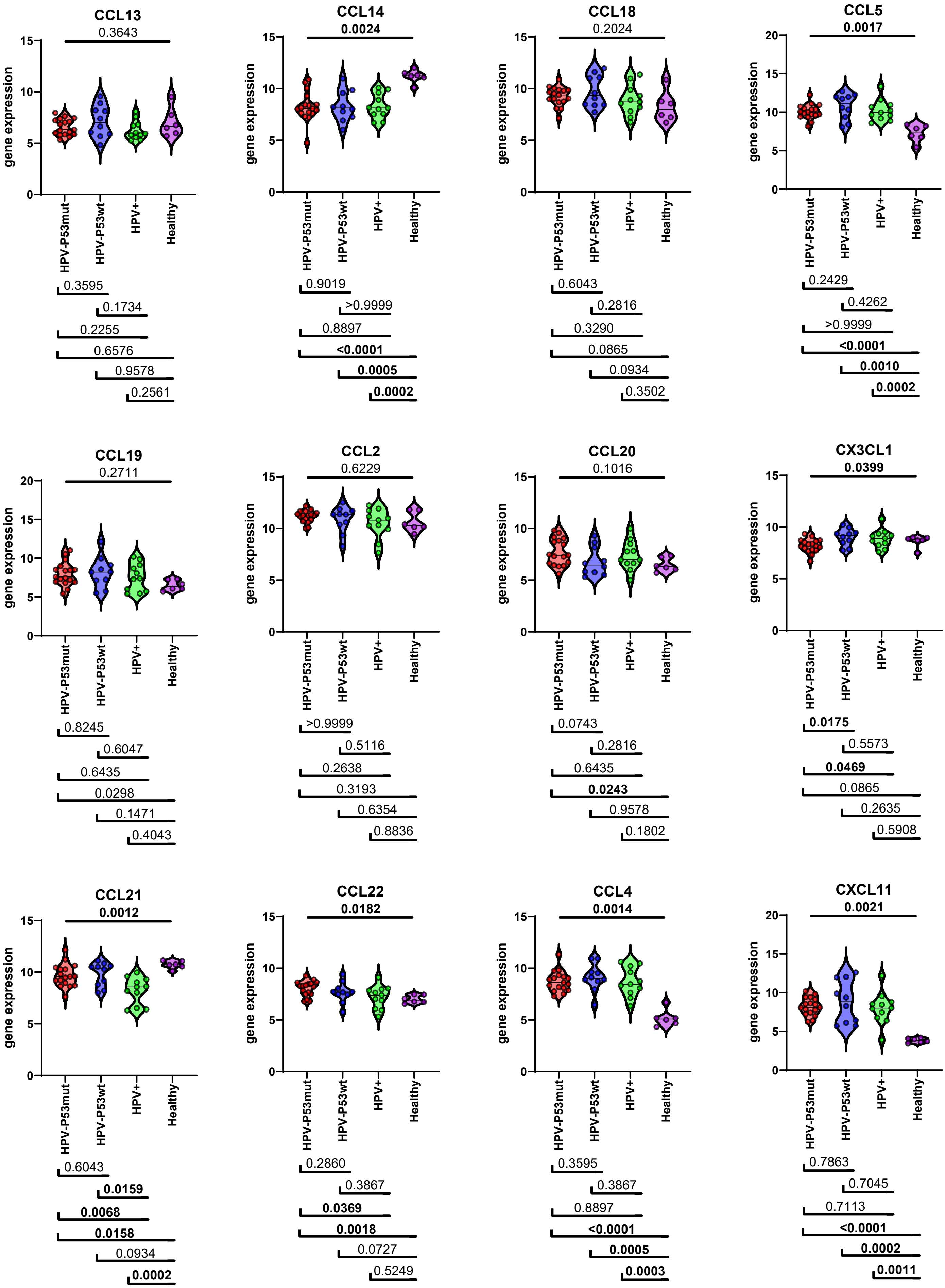

Supplemental Figure 6. Cytokine gene expression in VSCC, split per molecular subgroup, and in healthy vulva.
Violin plots for the expression of all cytokines by VSCC (total n=44) and in healthy HPV- vulva (n=6),
VSCC split per molecular subgroup: HPV-P53mut n=17, HPV-P53wt n=10, HPV+ n=11.
Kruskal Wallis test derived p-value depictend on top of violin plots, indicating a significant difference across the four groups.
Mann-Whitney U test derived p-values depicted below the violin plots, indicating a significant difference between two groups.

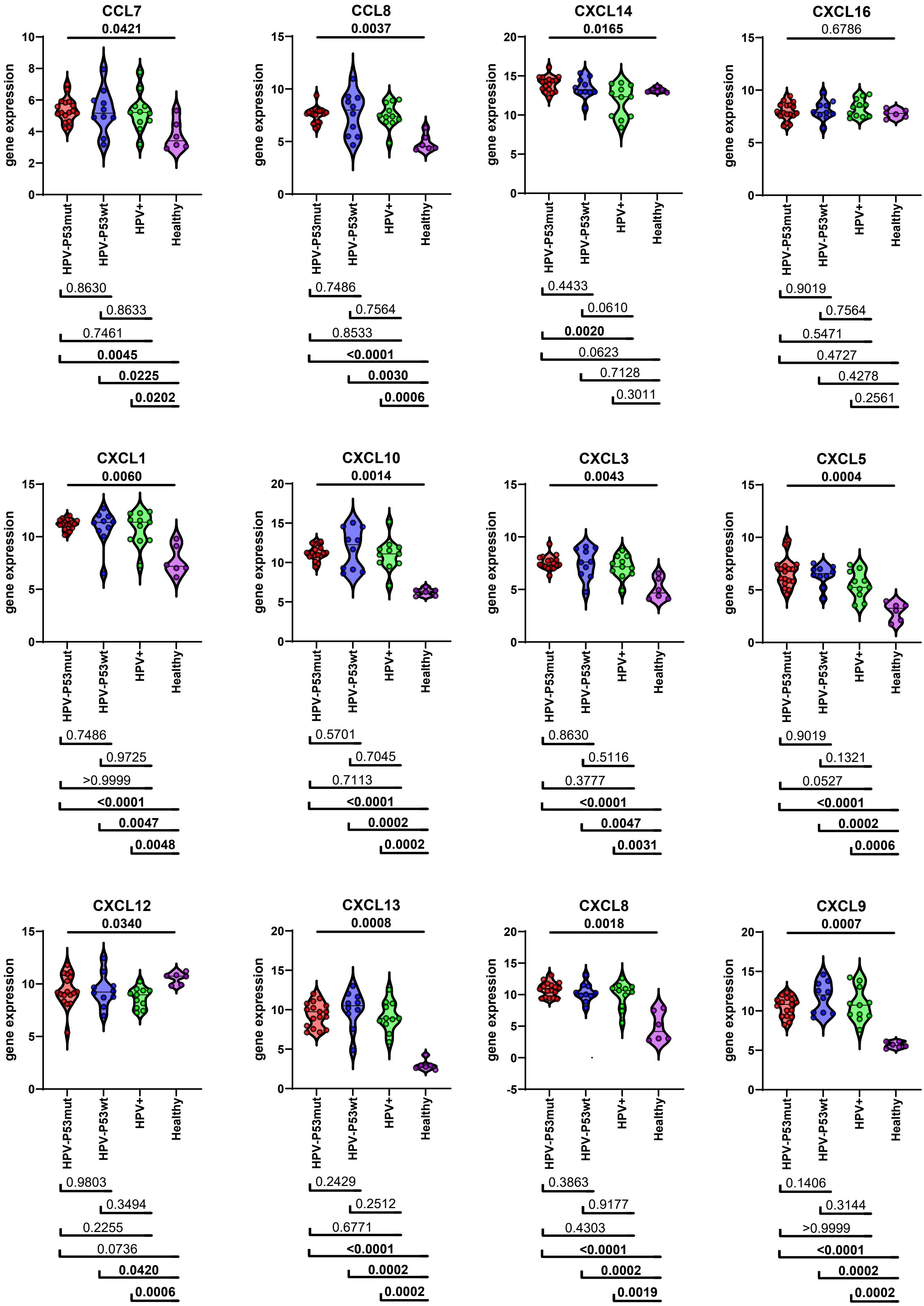
CCL7
0.0421
gene expression
HPV-P53mut
HPV-P53wt
HPV+
Healthy
0.8630
0.8633
0.7461
0.0045
0.0225
0.0202
CCL8
0.0037
gene expression
HPV-P53mut
HPV-P53wt
HPV+
Healthy
0.7486
0.7564
0.8533
<0.0001
0.0030
0.0006
CXCL14
0.0165
gene expression
HPV-P53mut
HPV-P53wt
HPV+
Healthy
0.4433
0.0610
0.0020
0.0623
0.7128
0.3011
CXCL16
0.6786
gene expression
HPV-P53mut
HPV-P53wt
HPV+
Healthy
0.9019
0.7564
0.5471
0.4727
0.4278
0.2561
CXCL1
0.0060
gene expression
HPV-P53mut
HPV-P53wt
HPV+
Healthy
0.7486
0.9725
>0.9999
<0.0001
0.0047
0.0048
CXCL10
0.0014
gene expression
HPV-P53mut
HPV-P53wt
HPV+
Healthy
0.5701
0.7045
0.7113
<0.0001
0.0002
0.0002
CXCL3
0.0043
gene expression
HPV-P53mut
HPV-P53wt
HPV+
Healthy
0.8630
0.5116
0.3777
<0.0001
0.0047
0.0031
CXCL5
0.0004
gene expression
HPV-P53mut
HPV-P53wt
HPV+
Healthy
0.9019
0.1321
0.0527
<0.0001
0.0002
0.0006
CXCL12
0.0340
gene expression
HPV-P53mut
HPV-P53wt
HPV+
Healthy
0.9803
0.3494
0.2255
0.0736
0.0420
0.0006
CXCL13
0.0008
gene expression
HPV-P53mut
HPV-P53wt
HPV+
Healthy
0.2429
0.2512
0.6771
<0.0001
0.0002
0.0002
CXCL8
0.0018
gene expression
HPV-P53mut
HPV-P53wt
HPV+
Healthy
0.3863
0.9177
0.4303
<0.0001
0.0002
0.0019
CXCL9
0.0007
gene expression
HPV-P53mut
HPV-P53wt
HPV+
Healthy
0.1406
0.3144
>0.9999
<0.0001
0.0002
0.0002

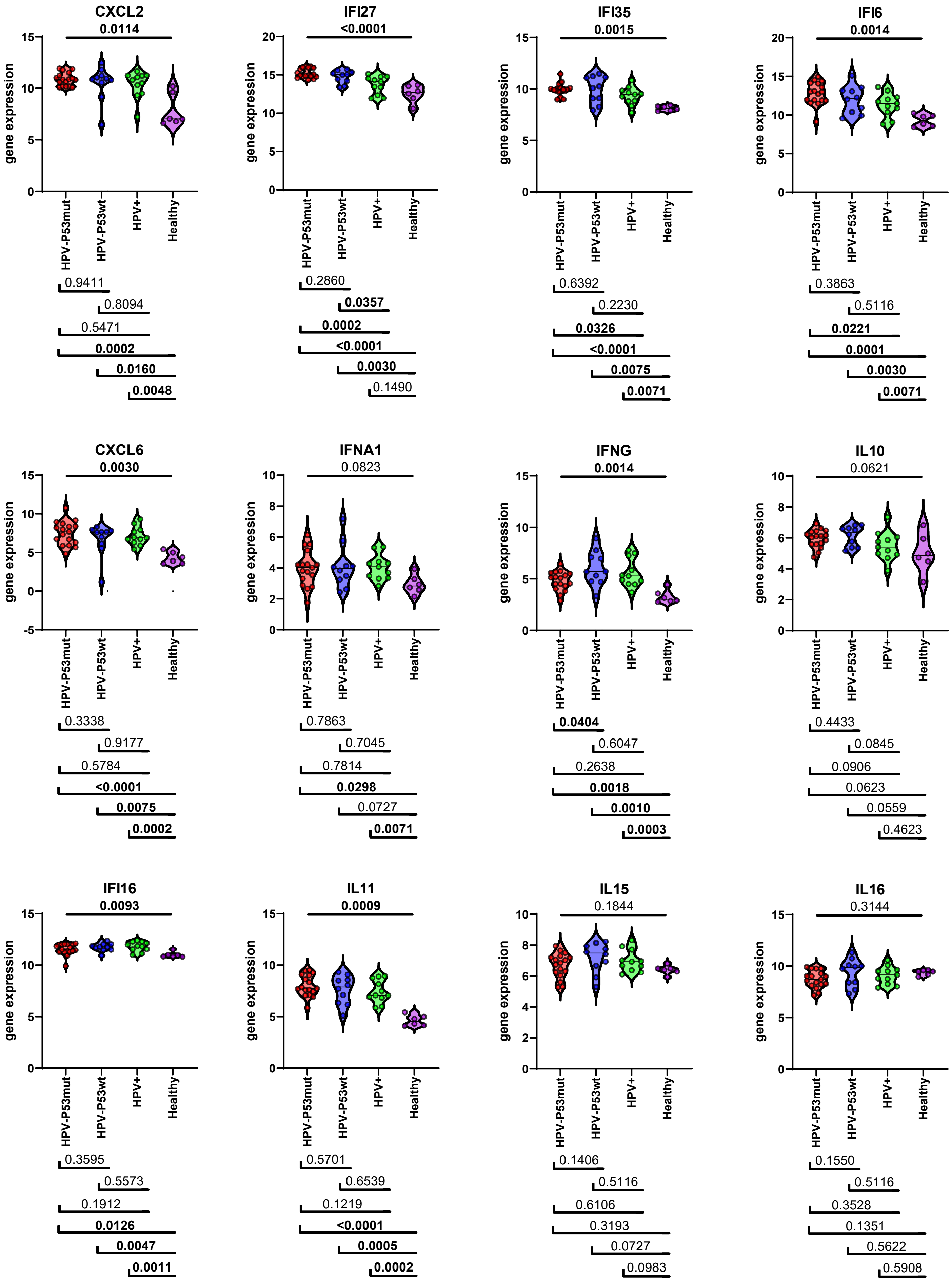
CXCL2
0.0114
gene expression
HPV-P53mut
HPV-P53wt
HPV+
Healthy
0.9411
0.8094
0.5471
0.0002
0.0160
0.0048
IFI27
<0.0001
0.2860
0.0357
0.0002
<0.0001
0.0030
0.1490
IFI35
0.0015
0.6392
0.2230
0.0326
<0.0001
0.0075
0.0071
IFI6
0.0014
0.3863
0.5116
0.0221
0.0001
0.0030
0.0071
CXCL6
0.0030
0.3338
0.9177
0.5784
<0.0001
0.0075
0.0002
IFNA1
0.0823
0.7863
0.7045
0.7814
0.0298
0.0727
0.0071
IFNG
0.0014
0.0404
0.6047
0.2638
0.0018
0.0010
0.0003
IL10
0.0621
0.4433
0.0845
0.0906
0.0623
0.0559
0.4623
IFI16
0.0093
0.3595
0.5573
0.1912
0.0126
0.0047
0.0011
IL11
0.0009
0.5701
0.6539
0.1219
<0.0001
0.0005
0.0002
IL15
0.1844
0.1406
0.5116
0.6106
0.3193
0.0727
0.0983
IL16
0.3144
0.1550
0.5116
0.3528
0.1351
0.5622
0.5908

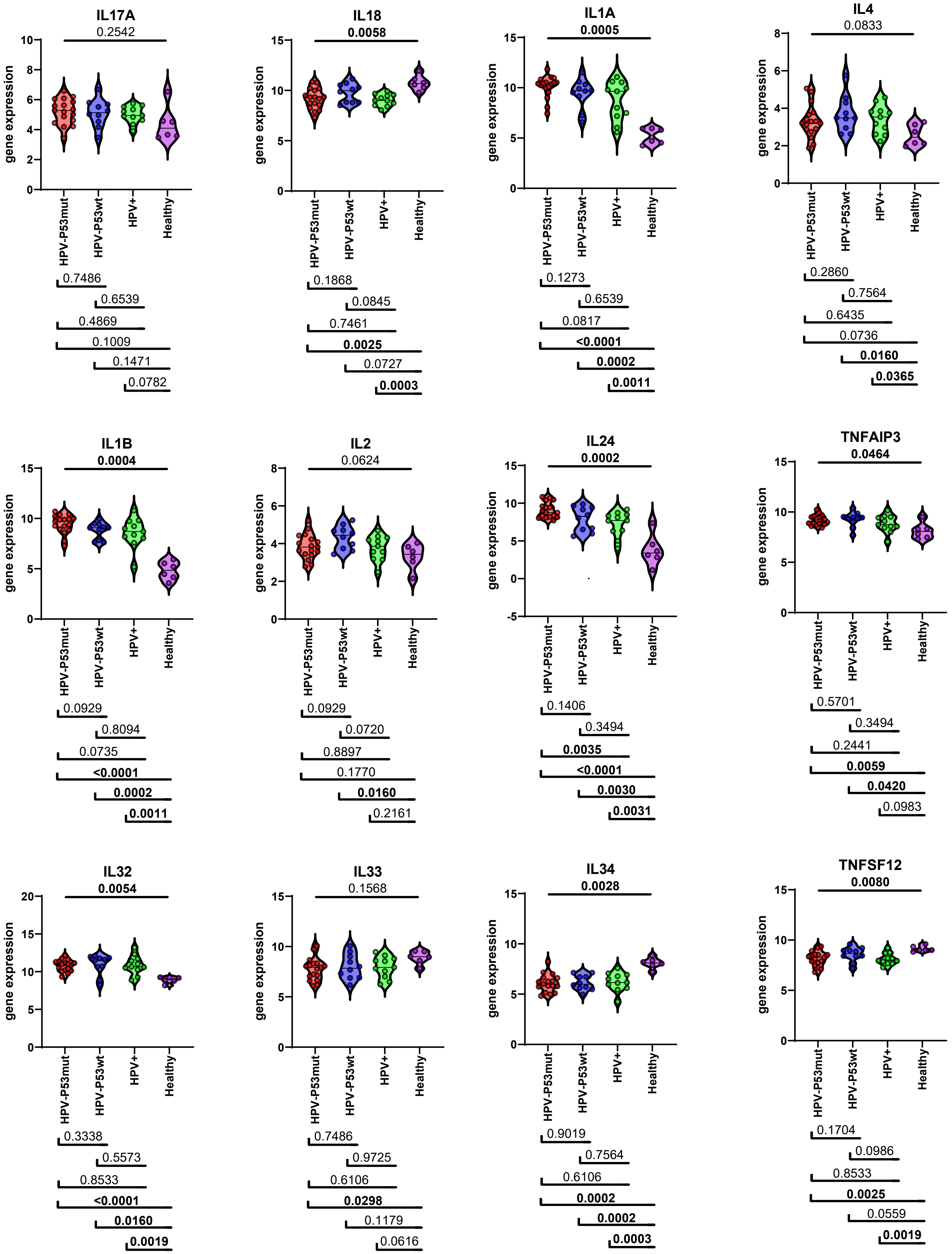
IL17A
0.2542
gene expression
HPV-P53mut
HPV-P53wt
HPV+
Healthy
0.7486
0.6539
0.4869
0.1009
0.1471
0.0782
IL18
0.0058
gene expression
HPV-P53mut
HPV-P53wt
HPV+
Healthy
0.1868
0.0845
0.7461
0.0025
0.0727
0.0003
IL1A
0.0005
gene expression
HPV-P53mut
HPV-P53wt
HPV+
Healthy
0.1273
0.6539
0.0817
<0.0001
0.0002
0.0011
IL4
0.0833
gene expression
HPV-P53mut
HPV-P53wt
HPV+
Healthy
0.2860
0.7564
0.6435
0.0736
0.0160
0.0365
IL1B
0.0004
gene expression
HPV-P53mut
HPV-P53wt
HPV+
Healthy
0.0929
0.8094
0.0735
<0.0001
0.0002
0.0011
IL2
0.0624
gene expression
HPV-P53mut
HPV-P53wt
HPV+
Healthy
0.0929
0.0720
0.8897
0.1770
0.0160
0.2161
IL24
0.0002
gene expression
HPV-P53mut
HPV-P53wt
HPV+
Healthy
0.1406
0.3494
0.0035
<0.0001
0.0030
0.0031
TNFAIP3
0.0464
gene expression
HPV-P53mut
HPV-P53wt
HPV+
Healthy
0.5701
0.3494
0.2441
0.0059
0.0420
0.0983
IL32
0.0054
gene expression
HPV-P53mut
HPV-P53wt
HPV+
Healthy
0.3338
0.5573
0.8533
<0.0001
0.0160
0.0019
IL33
0.1568
gene expression
HPV-P53mut
HPV-P53wt
HPV+
Healthy
0.7486
0.9725
0.6106
0.0298
0.1179
0.0616
IL34
0.0028
gene expression
HPV-P53mut
HPV-P53wt
HPV+
Healthy
0.9019
0.7564
0.6106
0.0002
0.0002
0.0003
TNFSF12
0.0080
gene expression
HPV-P53mut
HPV-P53wt
HPV+
Healthy
0.1704
0.0986
0.8533
0.0025
0.0559
0.0019

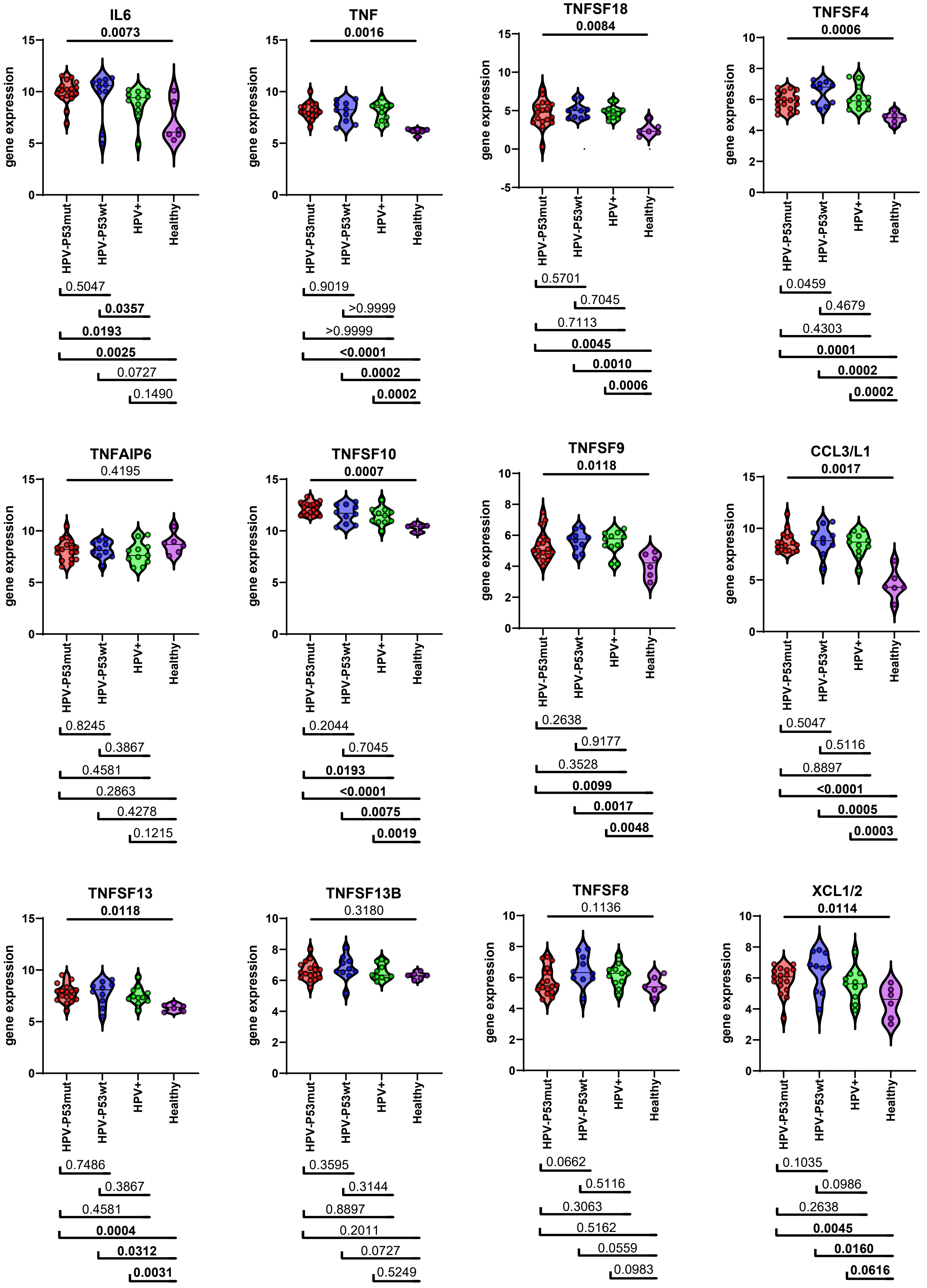

IL6
0.0073
gene expression
15
10
5
0
HPV-P53mut
HPV-P53wt
HPV+
Healthy
0.5047
0.0357
0.0193
0.0025
0.0727
0.1490
TNF
0.0016
gene expression
15
10
5
0
HPV-P53mut
HPV-P53wt
HPV+
Healthy
0.9019
>0.9999
>0.9999
<0.0001
0.0002
0.0002
TNFSF18
0.0084
gene expression
15
10
5
0
-5
HPV-P53mut
HPV-P53wt
HPV+
Healthy
0.5701
0.7045
0.7113
0.0045
0.0010
0.0006
TNFSF4
0.0006
gene expression
10
8
6
4
2
0
HPV-P53mut
HPV-P53wt
HPV+
Healthy
0.0459
0.4679
0.4303
0.0001
0.0002
0.0002
TNFAIP6
0.4195
gene expression
15
10
5
0
HPV-P53mut
HPV-P53wt
HPV+
Healthy
0.8245
0.3867
0.4581
0.2863
0.4278
0.1215
TNFSF10
0.0007
gene expression
15
10
5
0
HPV-P53mut
HPV-P53wt
HPV+
Healthy
0.2044
0.7045
0.0193
<0.0001
0.0075
0.0019
TNFSF9
0.0118
gene expression
10
8
6
4
2
0
HPV-P53mut
HPV-P53wt
HPV+
Healthy
0.2638
0.9177
0.3528
0.0099
0.0017
0.0048
CCL3/L1
0.0017
gene expression
15
10
5
0
HPV-P53mut
HPV-P53wt
HPV+
Healthy
0.5047
0.5116
0.8897
<0.0001
0.0005
0.0003
TNFSF13
0.0118
gene expression
15
10
5
0
HPV-P53mut
HPV-P53wt
HPV+
Healthy
0.7486
0.3867
0.4581
0.0004
0.0312
0.0031
TNFSF13B
0.3180
gene expression
10
8
6
4
2
0
HPV-P53mut
HPV-P53wt
HPV+
Healthy
0.3595
0.3144
0.8897
0.2011
0.0727
0.5249
TNFSF8
0.1136
gene expression
10
8
6
4
2
0
HPV-P53mut
HPV-P53wt
HPV+
Healthy
0.0662
0.5116
0.3063
0.5162
0.0559
0.0983
XCL1/2
0.0114
gene expression
10
8
6
4
2
0
HPV-P53mut
HPV-P53wt
HPV+
Healthy
0.1035
0.0986
0.2638
0.0045
0.0160
0.0616

A

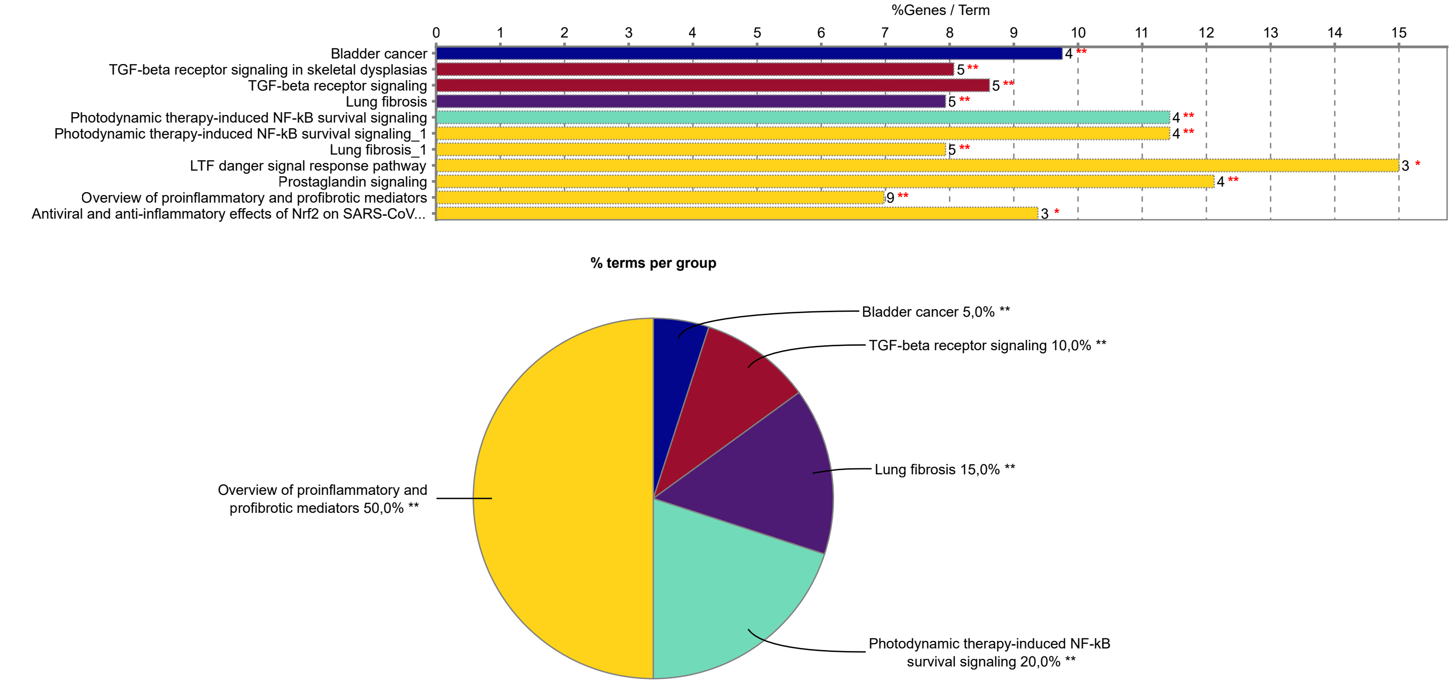

B

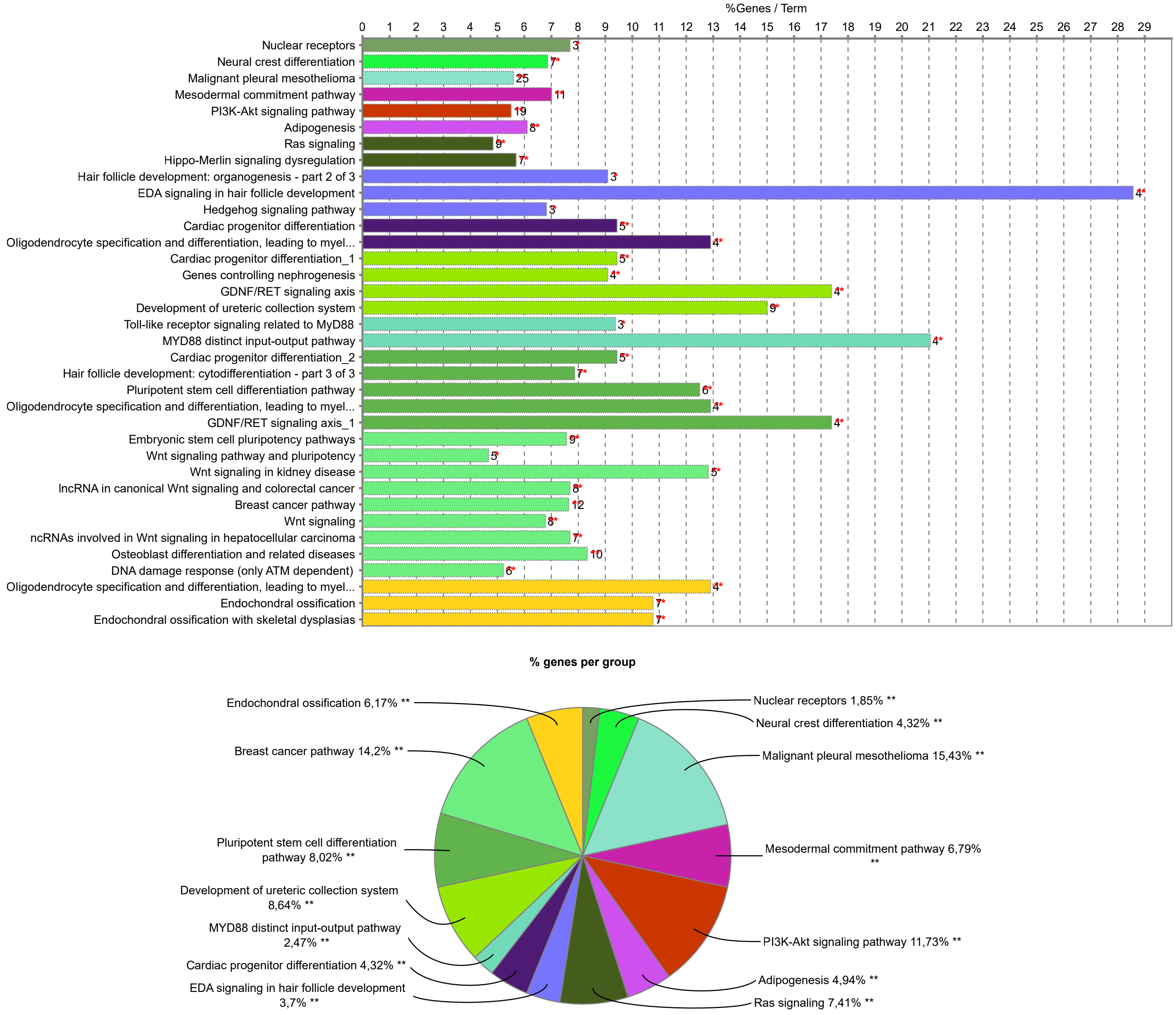

Supplemental Figure 7. Upregulated signaling pathways in monocyte hot and cold VSCC.
A) Epithelium monocyte hot (n=39) and in B) Epithelium monocyte cold (n=38) VSCC.

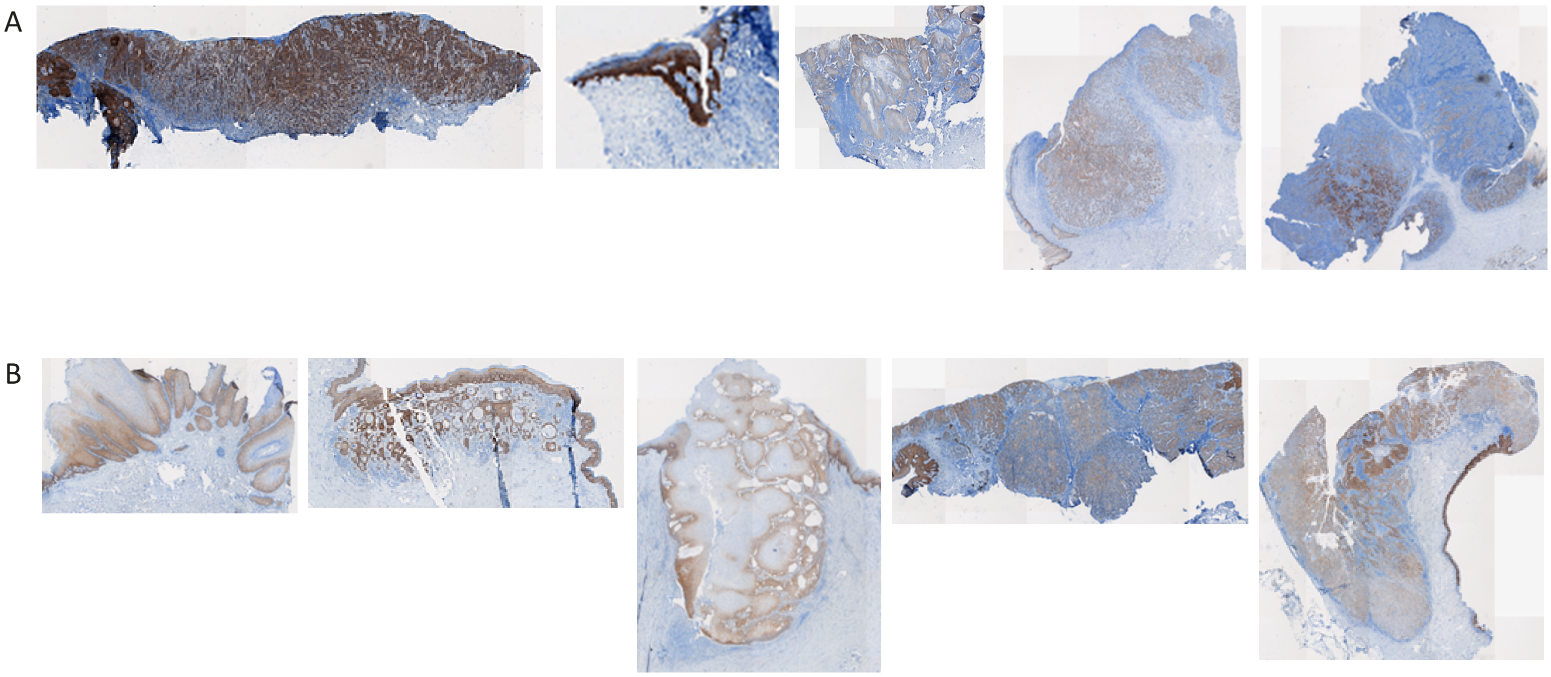

Supplemental Figure 8. Beta-catenin immunohistochemistry on myeloid hot versus cold VSCC.
10 representative cases of beta-catenin immunohistochemistry in A) epithelium myeloid hot (n=5) and B) epithelium myeloid cold (n=5) VSCC.

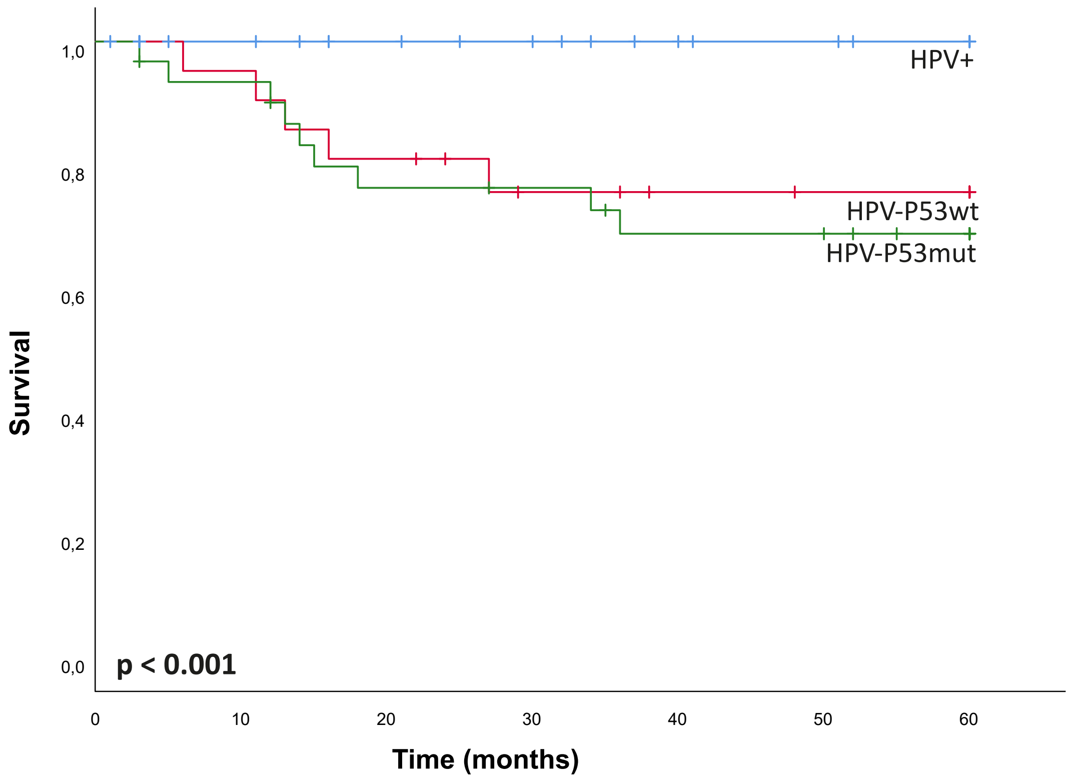

Supplemental Figure 9. Kaplan Meier survival curvers for VSCC, split per molecular subgroup.
VSCC total n=77. HPV-P53mut n=31, HPV-P53wt n=21, HPV+ n=25.
